# Chronic Low Back Pain with and without Concomitant Osteoarthritis: A Retrospective, Longitudinal Cohort Study of Patients in England

**DOI:** 10.1155/2023/5105810

**Published:** 2023-11-09

**Authors:** Greg Coates, Peter Clewes, Christoph Lohan, Hannah Stevenson, Robert Wood, Theo Tritton, Roger D. Knaggs, Alastair J. Dickson, David A. Walsh

**Affiliations:** ^1^Pfizer UK, Tadworth, UK; ^2^Pfizer Australia, Sydney, New South Wales, Australia; ^3^Adelphi Real World, Bollington, UK; ^4^Pain Centre Versus Arthritis and NIHR Nottingham Biomedical Research Centre, School of Medicine, University of Nottingham, Nottingham, UK; ^5^Primary Care Rheumatology & Musculoskeletal Medicine Society, York, UK; ^6^The North of England Low Back Pain Pathway, NIHR Applied Research Collaboration (ARC) North East and North Cumbria, St. Nicholas' Hospital, Newcastle Upon Tyne, UK; ^7^AD Outcomes Ltd., York, UK

## Abstract

**Objective:**

Despite the high prevalence of chronic low back pain (CLBP) and osteoarthritis (OA), few estimates of the economic cost of these conditions in England have been published. The aim of the present analysis was to characterise the economic burden of moderate-to-severe pain associated with CLBP + OA and CLBP alone compared with general population-matched controls without CLBP or OA. The primary objective was to describe the total healthcare resource use (HCRU) and direct healthcare costs associated with the target patient populations. Secondary objectives were to describe treatment patterns and surgical procedures.

**Methods:**

This was a retrospective, observational cohort study of patients receiving healthcare indicative of moderate-to-severe chronic pain associated with CLBP, with or without OA. We used linked longitudinal data from the Clinical Practice Research Datalink GOLD and Hospital Episode Statistics (HES). Patients (cases) were matched 1 : 1 with controls on age, sex, comorbidity burden, GP practice, and HES data availability.

**Results:**

The CLBP-alone cohort comprised 13 554 cases with CLBP and 13 554 matched controls; the CLBP + OA cohort comprised 7803 cases with both OA and CLBP and 7803 matched controls. Across all follow-up periods, patients with CLBP alone and those with CLBP + OA had significantly more GP consultations, outpatient attendances, emergency department visits, and inpatient stays than controls (all *p* < 0.0001). By 36 months after indexing, the mean (SD) per-patient total direct healthcare cost in the CLBP-alone cohort was £5081 (£5905) for cases and £1809 (£4451) for controls (*p* < 0.0001); in the CLBP + OA cohort, the mean (SD) per-patient total direct healthcare cost was £8819 (£7143) for cases and £2428 (£4280) for controls (*p* < 0.0001).

**Conclusion:**

Moderate-to-severe chronic pain associated with CLBP—with or without OA—has a substantial impact on patients and healthcare providers, leading to higher HCRU and costs versus controls among people with CLBP alone or together with OA.

## 1. Introduction

Lower back pain and osteoarthritis (OA) are common musculoskeletal conditions and major causes of chronic pain and disability in the UK. Chronic pain was reported by 34% of respondents in the 2017 Health Survey for England, with the most commonly reported being musculoskeletal, including the back, hips, and legs [1]. High-impact pain from musculoskeletal conditions, characterised by high pain intensity or severe limitation as a result of pain, was estimated to occur in 5.5 million people (12%) in England in 2021 [2]. Approximately one in six adults in England has some form of back pain [2]. The prevalence of back pain in England was estimated at 16.9% in 2012 [3]. Two additional studies reported prevalence estimates of 10.2% [4] and 11.0% [5], respectively, for chronic low back pain (CLBP) in England.

An estimated one in 10 adults in the UK has symptomatic, clinically diagnosed OA. [6] Between 2000/2001 and 2017/2018 over 3 million patients presented with OA in the secondary care setting in England [7]. The annual consultation incidence of OA in the primary care setting in England was 8.6 per 1000 men and 10.8 per 1000 women [8, 9].

Co-occurrence of low back pain (LBP) and OA is common. Gore and colleagues analysed data from patients who were newly prescribed selected pain medicines (nonsteroidal anti-inflammatory drugs (NSAIDs), cyclooxygenase-2 inhibitors, paracetamol, tramadol, and weak or strong opioids) in The Health Improvement Network UK database. They identified OA in 9.2–24.7% of patients with a diagnosis of CLBP and LBP in 7.4–21.9% of patients with a diagnosis of OA [10]. In a study of participants in the Progression Cohort of the US Osteoarthritis Initiative, 798 of 1389 patients (57%) with symptomatic OA of the knee had LBP [11]. More than a quarter (25.6%) of patients with LBP in an Australian population-based study had also been diagnosed with OA [12].

Management of pain due to CLBP or osteoarthritis is complex and generally includes a combination of pharmacological and nonpharmacological approaches, with varying results [13, 14]. Pharmacological management includes paracetamol and nonsteroidal anti-inflammatory drugs, and opioids have also been used, despite a lack of evidence of their efficacy [15, 16]. Nonpharmacological approaches to pain management include exercise, yoga, acupuncture, and meditation [17]. Upon progression of the disease to advanced joint destruction, surgery may play a part in patient management [18].

Despite the high prevalence of CLBP and OA, to our knowledge, there are few recent published estimates of the economic cost of these conditions in the UK, and existing evidence predates published guidance for the care and management of this patient population [19, 20]. The total cost of LBP management in the UK was estimated at £12.3 billion in 1998, comprising £1.6 billion in direct costs and £10.7 billion in indirect costs. [21] Analysis of the UK General Practice Research Database (now the Clinical Practice Research Datalink (CPRD)) reported that 12-month total healthcare costs for patients with CLBP were double those of matched controls during a study period spanning from 1^st^ January 2007 to 31^st^ December 2009 (£1074 vs £516; *p* < 0.05) [22]. General practitioner (GP) consultations accounted for 59% of the cost difference, 22% was for referrals to secondary care, and the rest was for analgesic medications [22]. Historically, the annual indirect cost of back pain was estimated at £10.7 billion in 2000 [21]. Existing literature provides little evidence on the cost of treating patients with chronic pain associated with LBP and on the cost of treating both OA and CLBP in the same individual. There is therefore a need for an up-to-date analysis of the economic burden of CLBP with and without concomitant OA in England.

The aim of the present analysis was to characterise the economic burden of patients in England receiving healthcare indicative of moderate-to-severe pain associated with CLBP + OA, and those with CLBP alone, compared with general population-matched controls without CLBP or OA.

## 2. Methods

### 2.1. Study Design and Patients

**Figure 1:**
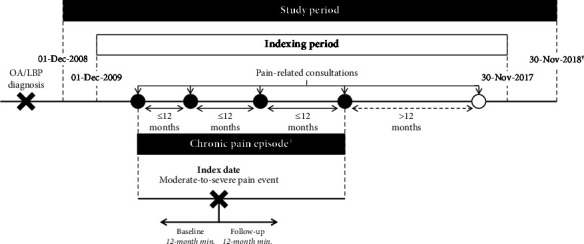
Study design. ^†^Latest available linked data at time of analysis. ^‡^Can start prior to the indexing and/or study period, but must start after the first LBP diagnosis code and continue into the indexing period. LBP, lower back pain; OA, osteoarthritis.

This retrospective, observational cohort study of patients presenting to primary care with an episode of moderate-to-severe chronic pain associated with CLBP, with or without OA, and matched controls without CLBP or OA was conducted using linked data from the CPRD and Hospital Episode Statistics (HES). The study design is shown in Figure 1. This methodology was previously used to describe resource use in a population with OA only and has been published elsewhere [9].

Patients with an existing diagnosis (Read code or International Statistical Classification of Diseases and Related Health Problems, 10th revision (ICD-10) code) of LBP, either with or without OA, were indexed between December 2009 and November 2017 on a moderate-to-severe pain event occurring within a period of chronic pain. To be included, patients were required to be aged ≥18 years at index, to be eligible for linkage to HES data, to have ≥12 months of data before indexing and ≥12 months of data after indexing, and to have data of acceptable research quality as defined by the CPRD (i.e., excluding patients with noncontinuous follow-up or patients with poor data recording that raises suspicion as to the validity of that patient's record).

A chronic pain episode was defined as a series of pain-related GP consultations relating to pain symptoms associated with CLBP or OA and/or a pain-related specialist consultation (rheumatology, orthopaedics, or pain management) in the secondary care setting, where gaps between visits were ≤12 months. The patient's first/earliest date of a moderate or severe pain event documented within the CPRD determined the index date.

Moderate-to-severe pain events were defined as any of the following: a referral to/attendance with a pain specialist; a surgical or nonsurgical invasive procedure relating to the treatment of CLBP or OA (procedures used routinely for reduction of pain were also included, even if not indicated for the treatment of CLBP); two or more prescriptions for NSAIDs, including at least two different NSAIDs or opioids within a 3-month period; or a pain-related emergency department (ED) visit, with a pain-related GP follow-up within 14 days.

Each patient within the study cohort (cases) was index matched 1 : 1 to a general population control within CPRD (without any current or past diagnosis of OA or LBP within their medical record) on year of birth (±1 year, e.g., a case born in 1980 could be matched to a control born between 1979 and 1981), sex, lifetime Charlson Comorbidity Index (CCI) score, GP practice, and linkage eligibility. The CCI is a score calculated based on the presence of a number of prespecified chronic conditions; the lifetime CCI score is the score calculated across the patient's entire medical record. Inclusion criteria and medical codes were reviewed for clinical relevance and appropriateness by RDK, AJD, and DAW. Each matched control patient was assigned a pseudoindex date equal to the index date of the case patient with whom they were matched.

### 2.2. Data Sources

The CPRD GOLD primary care database is a longitudinal, anonymised research database of computerised medical records held by GPs across the UK. Over 650 primary care practices in the UK, covering 11 million people, participate in the CPRD, with clinical records for over 12 million individuals; an estimated 5.5 million people are actively registered. Data are broadly representative of the UK population [23]. Available data in the CPRD include demographics, medical history (including diagnoses and health contacts), clinical investigation results, and prescribed medicines. Diagnostic data are recorded using Read codes, a coded thesaurus of clinical terms that has been in use since 1985. These provide a standard vocabulary for clinicians to record patient findings and procedures in health and social care systems across primary and secondary care.

Approximately 75% of English practices contributing to CPRD are linked to the HES dataset, which provides data on all inpatient and outpatient contacts, including ED visits, occurring at NHS hospitals in England, with diagnoses recorded using ICD-10 codes.

### 2.3. Study Objectives and Outcomes

The primary study objective was to describe total healthcare resource use (HCRU) and direct healthcare costs associated with the target patient populations (CLBP alone and CLBP + OA). Secondary study objectives were to describe CLBP/OA treatment patterns and surgical procedures.

The key study outcomes were HCRU and direct healthcare costs. Components of resource use included GP appointments, outpatient appointments, hospitalisations, ED attendances, medicine use, and total direct healthcare costs (the sum of all direct healthcare costs outlined above). Use of physiotherapy and other outpatient services was limited to services provided in the secondary care setting. Pharmacological management included nonopioid analgesics (paracetamol, systemic NSAIDs, topical NSAIDs, and other nonopioid analgesics), opioid analgesics (compound analgesics with weak opioids, weak opioids, and strong opioids), and adjuvant medicines (antidepressant, antiepileptic, anxiolytic/hypnotic agents). Pharmacological treatments assessed in this study were driven by the scope of NICE guidelines (NG59 and CG177) [19, 20], with the exception of adjuvant medicines, which are described in the British National Formulary [24]. HCRU was observed during 0–6, 0–12, and 0–24 months of follow-up after the index date). Costs were observed during 0–6, 0–12, 0–24, and 0–36 months of follow-up after the index date. Patients were included in each landmark analysis if they had sufficient follow-up data (i.e., patients needed at least 24 months of follow-up to be included in the 0–24 month analysis).

Unit cost data were transformed into direct healthcare costs using appropriate unit cost data. GP consultation costs were sourced from Unit Costs of Health and Social Care, compiled and provided by the Personal Social Services Research Unit [25]. For medicines prescribed in primary care, each identified product was matched to its listing in the NHS Drug Tariff [26, 27]. Healthcare resource groups (HRGs) were derived for each inpatient admission, outpatient attendance, and ED visit. These are standard groupings of clinically similar treatments/events that use common levels of healthcare resources and are derived for secondary care provision using the Local Payment Grouper. National prices and the national tariff workbook, compiled and provided by NHS Improvement and NHS England, were used to attach costs to each HRG [27].

### 2.4. Study Ethics

The study was conducted in accordance with legal and regulatory requirements and followed generally accepted research practices described in the Guidelines for Good Pharmacoepidemiology Practices issued by the International Society for Pharmacoepidemiology [28] and Good Practices for Outcomes Research issued by the International Society for Pharmacoeconomics and Outcomes Research [29–31].

This study was approved by the Independent Scientific Advisory Committee of the Medicines and Healthcare Products Regulation Agency (Protocol No. 19_158; approval date July 31^st^, 2019). Institutional review board approval was not required; no study participants were put at risk during the study, and confidentiality was maintained by use of data from deidentified electronic medical records provided by the CPRD.

### 2.5. Statistical Analysis

This was a retrospective analysis that was primarily descriptive in nature. This analysis was part of a larger study of the burden of moderate-to-severe chronic pain associated with OA or CLBP. The study was not designed to compare the CLBP + OA and CLBP-alone cohorts, but rather to describe each cohort with comparison to its matched controls. Base size, frequency, and percentages were reported for nominal variables; base size, mean, median, standard deviation (SD), 25th and 75th percentiles, minimum, and maximum values were reported for numeric variables.

All statistical tests were two-sided in nature; a significance level of *p* < 0.05 was used for comparison of cases and controls. No corrections were made for multiple comparisons.

Analyses were performed using Stata (version 16.1; StataCorp LLC, College Station, TX, USA).

## 3. Results

### 3.1. Patients

**Figure 2:**
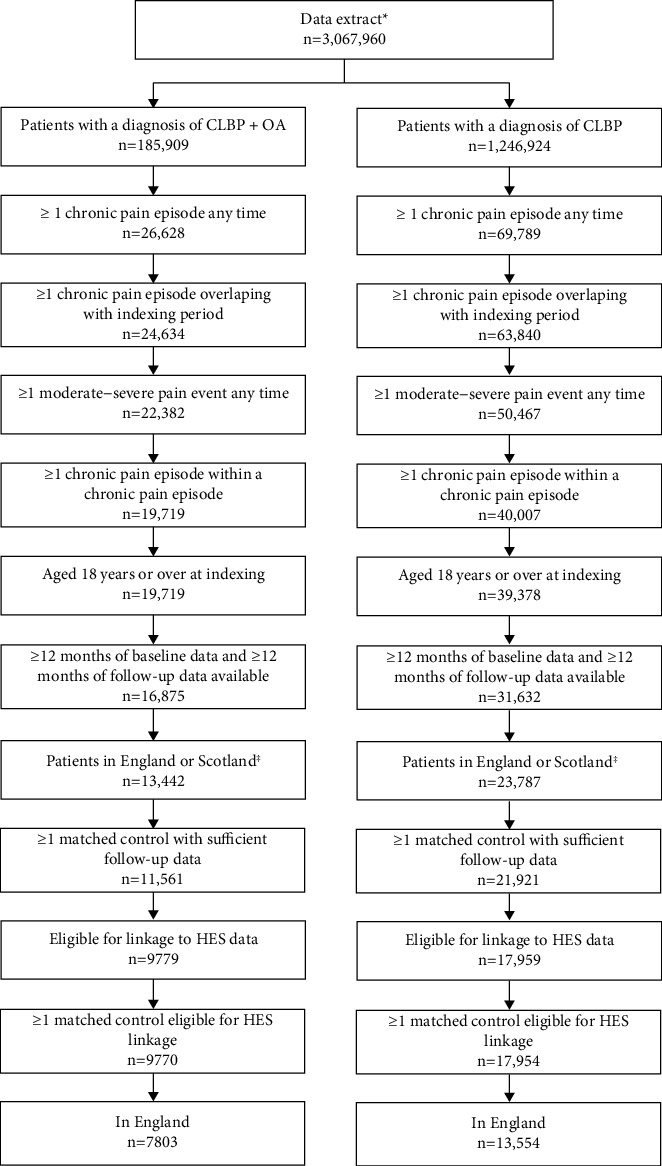
Study flowchart. ^†^Patients with at least one day of registration in the study period, aged 18 or older at the end of the indexing period, a medical record of acceptable research quality and at least one record of an OA and/or CLBP diagnosis code (Read or ICD-10) prior to study period end. ^‡^A separate analysis of Scottish patients was performed, the results of which are not presented here. CLBP, chronic low back pain; HES, hospital episode statistics; OA, osteoarthritis.

After applying the study inclusion and exclusion criteria, the CLBP-alone cohort comprised 13554 patients identified as having moderate-to-severe pain associated with CLBP between December 1^st^, 2008 and November 30^th^, 2018 (20.9% aged ≥65 years, 60.4% female), and an equal number of matched controls (Figure 2). Among these, 12090 were incident cases, i.e., with no moderate-to-severe chronic pain prior to indexing. The CLBP + OA cohort comprised 7803 patients with a diagnosis of both OA and CLBP (57.2% aged ≥65 years, 68.2% female), and an equal number of matched controls (Table 1); 7520 of these cases were incident cases.

Patient demographic and clinical characteristics are shown in Table 1. Although cases and controls were matched on the lifetime CCI score as well as other factors, both the CLBP-alone and CLBP + OA cases had statistically significantly higher prevalence estimates for several comorbid physical conditions prior to indexing than controls, including hypertension, hyperlipidaemia, asthma, and diabetes (all *p* < 0.0001). Not all of these comorbidities were included in the CCI, upon which cases and controls were matched. Comorbid mental health conditions, including anxiety and depression, were also more common in cases compared with controls (all *p* < 0.05 vs controls). The mean length of follow-up was 43.6 months in the CLBP-alone cohort and 39.9 months in the CLBP + OA cohort.

### 3.2. Healthcare Resource Utilisation

#### 3.2.1. CLBP Alone

Across all follow-up periods (0–6, 0–12, 0–24, and 0–36 months), patients with CLBP alone had significantly more GP consultations, outpatient attendances, ED visits, and inpatient stays compared with matched controls (all *p* < 0.0001) (Table 2). Orthopaedics was the most frequently used pain-related outpatient service during the first 12 months of follow-up (38.2% of cases vs 3.5% of controls; *p* < 0.0001); 26.7% of cases used pain management outpatient services during the same period compared with 0.2% of controls (*p* < 0.0001; Supplementary Table 1). The cumulative inpatient length of stay was approximately twice as long for cases compared with controls in the first 12 months of follow-up (mean (SD) 1.46 (8.38) vs 0.60 (5.69) days; *p* < 0.0001). Similar findings were observed during 0–6, 0–24, and 0–36 months of follow-up (Table 2).

By 36 months after indexing, cases had a mean (SD) of 35.09 (26.94) GP consultations, 13.12 (14.44) outpatient attendances, 1.44 (2.91) ED visits, and 2.22 (4.57) inpatient stays compared with 14.32 (17.61), 4.15 (8.69), 0.56 (1.17), and 0.66 (5.62), respectively, in controls (all *p* < 0.0001). The mean (SD) length of stay over the 36-month follow-up was 0.82 (2.86) days in cases and 0.58 (4.49) days in controls (*p* < 0.0001).

The median (25^th^ and 75^th^ percentiles) number of treatment lines received was 9.0 (5−18), and the mean (SD) was 13.1 (12.3). Among cases, prescribing of paracetamol (9.9–20.9%) and topical NSAIDs (4.9–8.8%) increased substantially from line 1 to 10 (Supplementary Figure 1A), whereas the prescribing of systemic NSAIDs (30.4–30.6%), weak opioids (11.4–10.4%), and strong opioids (27.7–32.1%) remained relatively constant. Use of antidepressants (tricyclics, selective serotonin reuptake inhibitors, and serotonin and norepinephrine reuptake inhibitors; 12.9–30.6%), the antiepileptic agents pregabalin and gabapentin (8.2–21.4%), and anxiolytics (9.7–16.4%) increased substantially from line 1 to 10.

Among the 12090 incident cases with moderate-to-severe chronic pain associated with CLBP prior to indexing, 169 (1.4%) had fusion surgery, 248 (2.1%) had disc replacement surgery, 1692 (14.0%) had medial branch block, and 334 (2.8%) had radiofrequency denervation.

#### 3.2.2. CLBP + OA

Across all follow-up periods (0–6, 0–12, 0–24, and 0–36 months), patients with CLBP + OA had significantly more GP consultations, outpatient attendances, ED visits, and inpatient stays compared with matched controls (all *p* < 0.0001) (Table 3).

As with the CLBP-alone cohort, patients were most frequently referred to the orthopaedics outpatient service for their pain during the first 12 months of follow-up (65.0% of cases vs 3.8% of controls; *p* < 0.0001); 18.8% of cases used the pain management outpatient service during the same period compared with 0.3% of controls (*p* < 0.0001; Supplementary Table 2).

The cumulative inpatient length of stay was almost three times longer for cases compared with controls in the first 12 months of follow-up (mean (SD) 2.52 (9.03) vs 0.90 (7.13) days; *p* < 0.0001).

Similar findings were observed during 0–6, 0–24, and 0–36 months of follow-up (Table 3). By 36 months after indexing, cases had a mean (SD) of 44.44 (28.58) GP consultations, 19.77 (17.31) outpatient attendances, 1.50 (2.81) ED visits, and 3.06 (3.23) inpatient stays compared with 19.00 (18.74), 5.70 (10.78), 0.55 (1.09), and 0.96 (8.32), respectively, in controls. The mean (SD) length of stay over the 24-month follow-up was 1.69 (5.11) days in cases and 0.90 (4.38) days in controls.

The median (25^th^ and 75^th^ percentiles) number of treatment lines received was 11.0 (6–20), while the mean (SD) was 14.8 (12.5). Prescribing of paracetamol (19.2–32.3%), topical NSAIDs (9.1–15.6%) increased substantially from line 1 to 10 in cases with OA + CLBP, whereas the prescribing of systemic NSAIDs (23.8–27.9%), weak opioids (11.3–11.3%), and strong opioids (26.3–30.3%) remained relatively consistent. Use of antidepressants (12.3–26.5%), antiepileptics (pregabalin and gabapentin; 7.5–18.8%), and anxiolytics (6.5–13.3%) increased substantially from line 1 to 10 (Supplementary Figure 1B).

Among the 7520 incident cases, i.e., those without any moderate-to-severe chronic pain associated with CLBP prior to indexing, 1844 (24.5%) had a total joint replacement (knee, *N* = 993; hip, *N* = 861; other, *N* = 84), 197 (2.6%) had fusion surgery, 188 (2.5%) had arthroscopy (knee, *N* = 1.5; hip, *N* = 18; other, *N* = 54), 122 (1.6%) underwent osteotomy (knee, *N* = 2; hip, *N* = 1; other, *N* = 120), and 70 (0.9%) had disc-replacement surgery. A further 698 cases (9.3%) had a medial branch block, 150 (2.0%) had radiofrequency denervation, and 2044 (27.2%) had an intraarticular injection.

### 3.3. Costs

#### 3.3.1. CLBP Alone

**Figure 3:**
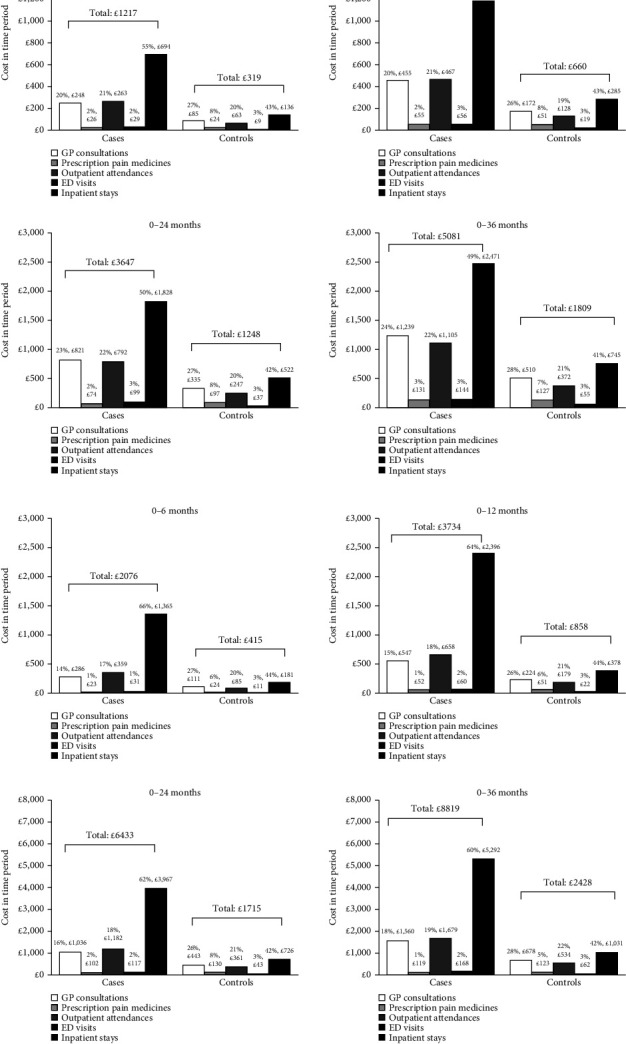
Direct healthcare costs in the 6, 12, 24, and 36 months following indexing in (a) patients with CLBP and controls and (b) patients with CLBP + OA and controls. Not all cases/controls had 24 and 36 months of follow-up. *p* < 0.0001 for the difference in mean values between cases and controls for ED visits, GP consultations, outpatient attendances, inpatient stays and total costs, across all time periods. ED, emergency department; CLBP, chronic low back pain; GP, general practitioner; OA, osteoarthritis.

NHS England total healthcare costs incurred through cases during the 0–6, 0–12, 0–24, and 0–36 months of follow-up were 4, 3.5, 3, and 2.8 times higher, respectively, compared with matched controls (all *p* < 0.0001) (Figure 3(a)). By 36 months after indexing, the mean (SD) total direct healthcare cost per patient had risen to £5081 (£5905) for cases and £1809 (£4451) for controls (*p* < 0.0001). Individual cost elements for each healthcare resource used were also significantly higher for cases compared with controls. The contribution of inpatient stays to total costs was higher for cases (49–55%) versus controls (41–43%) across all time periods. GP consultations and outpatient attendances were other substantial contributors to the total costs (20–23% and 21-22%, respectively, in cases; 26-27% and 19–21% in controls, respectively).

Medication costs were numerically lower in cases versus controls. They accounted for 2-3% of total costs in cases (£26 of £1271 at 0−6 months to £131 of £5081 at 0−36 months) and 7-8% of total costs in controls (£24 of £319 at 0−6 months to £127 of £1809 at 0−36 months).

#### 3.3.2. CLBP + OA

Total costs incurred by cases during the 0–6, 0–12, 0–24, and 0–36 months of follow-up were 5, 4.4, 3.8, and 3.6 times higher, respectively, compared with matched controls (all *p* < 0.0001) (Figure 3(b)). By 36 months after indexing, the mean (SD) total direct healthcare cost per patient was £8819 (£7143) for cases and £2428 (£4280) for controls (*p* < 0.0001). Individual cost elements for HCRU were significantly higher for cases compared with controls.

Inpatient stay costs accounted for approximately 60−66% of total costs for cases and 42−44% for controls (*p* < 0.0001). GP consultations and outpatient attendances were other substantial contributors to the total costs (14–17% and 17–19%, respectively, in cases; 26-27% and 20–22% in controls).

Medication costs were numerically lower in cases versus controls across all time periods, accounting for 1-2% of the total costs in cases (£23 of £1365 at 0–6 months and £119 of £8818 at 0–36 months) versus 6–8% of the total cost in controls (£24 of £415 at 0–6 months to £123 of £2428 at 0−36 months).

## 4. Discussion

This retrospective, longitudinal cohort study with matched controls is, to our knowledge, the first to provide a detailed analysis of HCRU and costs incurred by patients in England who have CLBP, with or without OA, and who experience moderate-to-severe pain. A total of 13734 patients with moderate-to-severe chronic pain associated with CLBP were identified, of whom 7803 (57%) also had a diagnosis of OA. Moreover, we identified 21357 patients with moderate-to-severe chronic pain associated with CLBP, of whom 7803 (37%) also had a diagnosis of OA. These estimates of OA as a comorbidity in patients with CLBP are higher than reported elsewhere [10, 12], most likely as a result of the selection of patients with moderate-to-severe pain.

Patients with moderate-to-severe pain associated with CLBP alone used significantly more healthcare services and incurred greater direct healthcare costs than their age-, sex-, geography-, and comorbidity-matched controls. The total direct cost of treating this patient cohort over 36 months was approximately £35 million (mean direct cost per patient of £5081 over 36 months), a considerable cost bearing in mind the relatively small size of the patient cohort (6724 patients with data at 36 months). Importantly, these direct costs represent a small proportion of the overall cost associated with CLBP, as our analysis did not take into consideration indirect costs, such as sickness absence and reduced productivity, and the cost of informal care, such as self-care and family care, not provided by the NHS. Inpatient stays were the main drivers of costs incurred in this patient population.

Similarly, patients with moderate-to-severe pain associated with CLBP with concomitant OA used significantly more healthcare services and incurred greater direct healthcare costs than their matched controls. The total direct cost of treating these patients over 36 months was almost £31 million, with each patient incurring costs that were almost twice as much as those incurred by a patient with CLBP alone, primarily driven by inpatient stays and outpatient appointments. Almost one-quarter of patients in the CLBP + OA cohort underwent total joint replacement during the follow-up period, most commonly knee and hip replacement, considerably adding to the cost of their management. A small proportion of patients in both the CLBP-alone and CLBP + OA cohorts underwent procedures that were not recommended in the most recent guidelines for CLBP management (NICE low back pain and sciatica treatment guidelines published in 2016 and updated in 2020 [19]). Use of surgical interventions such as spinal fusion and disc replacement might suggest that patients had exhausted other treatment options and indicate the considerable financial cost of moderate-to-severe pain and its management in patients affected by two of the most common chronic conditions in the UK. These data also indicate the cost savings to the NHS that might be achieved by applying the most recent evidence-based guidelines for CLBP and avoiding referring patients for nonrecommended treatments. Our data may also be applicable to the NHS England Best MSK Health Collaborative, an initiative aiming to reduce variations in access outcomes and experiences [32].

Others have reported similar HCRU trends in patients with CLBP compared with matched controls. In a US study of data from a managed care system, Mapel and colleagues observed that patients with CLBP were three times more likely to be hospitalised and had longer average lengths of stay—incurring greater costs—than their matched controls [33]. In an analysis of total direct healthcare costs in patients with CLBP in the UK, Hong and colleagues reported that costs were twice as high in patients with CLBP as in matched controls [22]. We observed that costs in our cases were approximately three times those of controls, most likely a reflection of greater pain severity in our cohort of patients with moderate-to-severe pain compared with the earlier analysis in which pain severity was not prespecified [22].

The use of some analgesic medicines, including paracetamol and topical NSAIDs, increased across treatment lines in both cohorts of cases. Use of strong opioids remained consistent across lines in the CLBP cohort, at approximately one-third of patients. Since 2016, NICE guidelines for managing pain in people with chronic LBP advise against the use of most opioids, whereas the 2020 update to the OA management guidelines recommend opioids use with caution [19, 20]. Notably, the use of antiepileptics also increased substantially across treatment lines, suggesting that when commonly used analgesic medicines failed to control symptoms, physicians were perhaps resorting to alternative agents with limited evidence of efficacy and potential for adverse events. Antidepressants and anxiolytic medicines were more commonly used in later versus earlier lines of therapy, possibly reflecting an increase in anxiety and depression in both cohorts of cases, that other treatment options were already being used, or that the patient's pain had a higher impact and thus required additional medication. Anxiety and depression were significantly more prevalent in cases than controls at indexing, with 35% of cases with CLBP only and 36% of those with CLBP + OA classified as having depression. Mental health conditions are known to be comorbid with chronic pain. Carstenen and colleagues observed that the largest comorbidity subgroup in their study of Swedish people was the combination of back pain and depression [34], and Xu and colleagues reported that mental health disorders were common in Chinese people with chronic back or neck pain [35].

Some limitations of the study, many of which are inherent in real-world data analyses, should be considered. Diagnoses were identified using ICD-10 and Read codes, which are subject to potential miscoding. Secondary data sources are liable to have incomplete, missing, or low-quality data; the absence of a specific diagnosis or drug code was required to be interpreted as the absence of disease and medicines, respectively, resulting in potential misclassification bias. The CPRD is, however, widely considered a gold standard in healthcare event reporting, and missed diagnoses or prescriptions are likely to be rare. The requirement for 12 months of follow-up data following the index date introduces potential survivorship bias to the study. The chronic pain end date may not truly indicate the date when the chronic pain episode ended, as some patients may instead have decided not to use the NHS further and/or self-manage despite minimal improvement (if any) in their chronic pain.

Not all HRGs have a national tariff, as some prices are negotiated locally; therefore, direct healthcare costs are likely to have been underestimated in this analysis as missing costs were not imputed. Our data were captured before changes in the recommended pharmacological treatment for people with LBP, specifically the use of opioids, and therefore might not reflect current pain management practice. Medicines bought over the counter, such as paracetamol and some NSAIDs, which have been reported to account for an annual spend of £600 million, and those administered through hospital pharmacies are not captured, and there is no national individual-level hospital prescribing database for England [36]. This may explain the relative lack of difference between cases and controls in medicine costs.

Some analgesic medications have multiple indications; therefore, it is unknown if the medicine was prescribed to specifically treat CLBP or OA pain, or other pain and nonpain conditions. The definition of the study cohort and the involvement of clinical experts in the development of the protocol and statistical analysis plan meant that analgesics were likely to have been prescribed to treat the chronic pain associated with CLBP. The costs described in this study represent direct costs only; additional self-care and private care costs, plus societal and indirect employment costs, were not captured in this analysis but are likely to substantially increase the overall cost of chronic pain associated with CLBP.

A key study strength is the real-world nature of the data. The linked CPRD and HES datasets have broad coverage, encompassing 75% of patients in English GP practices. Consequently, our findings are likely to be generalisable as an accurate representation of adults with CLBP alone and adults with OA + CLBP in England.

## 5. Conclusions

Moderate-to-severe chronic pain associated with CLBP with or without OA has a substantial impact on patients and healthcare providers. We have shown that CLBP and OA are commonly comorbid and that healthcare utilisation and costs are increased for people with CLBP alone or comorbid with OA. This increase in healthcare utilisation and costs is largely associated with hospital admissions and outpatient attendances. Current costs for analgesic medicines are relatively low, but improvements in medical management have substantial potential to reduce resource utilisation if they can abrogate the need for more intensive care. Future research may look to describe resource use in patients with mild chronic pain and CLBP, to explore the indirect economic burden in this patient population. Further, with the establishment of integrated care boards (ICBs) and as the NHS becomes more data-driven, opportunities for similar research to use data derived from a broader breadth of aspects of care, such as from hospital pharmacies, may arise.

## Figures and Tables

**Table 1:** Demographic and clinical characteristics of cases and general population controls.

Characteristic	CLBP alone		CLBP + OA	
	Cases (*N* = 13 554)	Controls (*N* = 13 554)	Cases (*N* = 7803)	Controls (*N* = 7803)
*Age at indexing* ^*†*^ *, years*				
Mean (SD)	50.4 (16.0)	50.4 (16.0)	66.1 (11.9)	66.1 (11.9)
≥65 years, *n* (%)	2838 (20.9)	2838 (20.9)	4463 (57.2)	4463 (57.2)

*Sex* ^*†*^ *, n (%)*				
Male	5374 (39.6)	5374 (39.6)	2478 (31.8)	2478 (31.8)
Female	8180 (60.4)	8180 (60.4)	5325 (68.2)	5325 (68.2)
Mean BMI, kg/m^2^ (SD)	29.2 (6.3)^‡^	—	30.6 (6.1)^§^	—

*Comorbid physical conditions* ^*‡*^ *, n (%)*				
Hypertension	3480 (25.7)^*∗∗∗*^	2766 (20.4)	4225 (54.1)^*∗∗∗*^	3154 (40.4)
Asthma	2621 (19.3)^*∗∗∗*^	2192 (16.2)	1747 (22.4)^*∗∗∗*^	1232 (15.8)
Hyperlipidaemia	1818 (13.4)^*∗∗∗*^	1125 (8.3)	2333 (29.9)^*∗∗∗*^	1286 (16.5)
Diabetes	1031 (7.6)^*∗∗∗*^	772 (5.7)	1073 (13.8)^*∗∗∗*^	831 (10.6)
Other CHD	802 (5.9)^*∗∗∗*^	469 (3.5)	1109 (14.2)^*∗∗∗*^	655 (8.4)

*Comorbid mental health conditions, n (%)*				
Depression	4785 (35.3)^*∗∗∗*^	1745 (12.9)	2824 (36.2)^*∗∗∗*^	969 (12.4)
Anxiety	2350 (17.3)^*∗∗∗*^	888 (6.6)	1451 (18.6)^*∗∗∗*^	613 (7.9)
Mean CCI, lifetime value (SD)^†^	0.83 (1.33)	0.83 (1.33)	1.34 (1.60)	1.34 (1.60)

*Length of follow-up, months*				
Mean (SD)	43.6 (22.8)	43.0 (22.5)	39.9 (20.5)	39.1 (20.3)
Mean time from diagnosis to indexing, years (SD)	5.4 (5.3)^§^	NA	4.0 (4.3)^¶^	NA

^†^Used to match cases to controls. ^‡^*N* = 5429 due to missing data. ^§^*N* = 4044 due to missing data. ^¶^Top five comorbidities recorded prior to indexing (from a prespecified list; not all possible comorbid conditions were assessed). ^§^First instance of LBP diagnostic code within the patient's medical record. ^¶^Later of first instance of an OA or LBP diagnosis in the patient's medical record. ^*∗*^*p* < 0.05; ^*∗∗∗*^*p* < 0.0001 versus controls. Abbreviations: BMI, body mass index; CCI, Charlson Comorbidity Index; CHD, coronary heart disease; CLBP, chronic lower back pain; GP, general practitioner; LBP, lower back pain; NA, not applicable; OA, osteoarthritis; SD, standard deviation.

**Table 2:** Healthcare resource utilisation in patients with chronic low back pain alone and matched controls.

Healthcare service use	0–6 months		0–12 months		0–24 months^†^		0–36 months^†^	
	Cases (*N* = 13 554)	Controls (*N* = 13 554)	Cases (*N* = 13 554)	Controls (*N* = 13 554)	Cases (*N* = 9728)	Controls (*N* = 9728)	Cases (*N* = 6724)	Controls (*N* = 6734)
*GP consultation*								
Mean (SD)	7.39 (5.85)	2.45 (3.54)	13.54 (10.38)	4.94 (6.52)	24.36 (18.52)	9.56 (12.38)	35.09 (26.94)	14.32 (17.61)
Median (IQR)	6 (3–10)	1 (0–3)	11 (7–17)	3 (1–7)	20 (12–31)	7 (2–13)	29 (18–45)	10 (4–19)

*Outpatient attendance*								
Mean (SD)	3.05 (3.69)	0.71 (2.24)	5.61 (6.34)	1.44 (3.84)	9.53 (10.80)	2.80 (6.35)	13.12 (14.44)	4.15 (8.69)
Median (IQR)	2 (1–4)	0 (0–0)	4 (1–8)	0 (0–1)	6 (3–13)	0 (0–3)	9 (4–18)	1 (0–5)

*ED visit*								
Mean (SD)	0.30 (0.89)	0.10 (0.43)	0.57 (1.55)	0.20 (0.67)	1.00 (2.14)	0.38 (0.95)	1.44 (2.91)	0.56 (1.17)
Median (IQR)	0 (0–0)	0 (0–0)	0 (0–1)	0 (0–0)	0 (0–1)	0 (0–0)	1 (0–2)	0 (0–1)

*Inpatient stay*								
Mean (SD)	0.56 (1.28)	0.11 (0.94)	1.00 (2.50)	0.24 (2.02)	1.62 (3.87)	0.46 (3.83)	2.22 (4.57)	0.66 (5.62)
Median (IQR)	0 (0-1)	0 (0-0)	1 (0-1)	0 (0-0)	1 (0–2)	0 (0-0)	1 (0–3)	0 (0-1)

*Length of stay, days* ^*‡*^								
Mean (SD)	0.86 (5.35)	0.29 (3.90)	1.46 (8.38)	0.60 (5.69)	2.12 (10.60)	1.20 (10.30)	2.83 (11.80)	1.43 (9.99)
Median (IQR)	0 (0-0)	0 (0-0)	0 (0-0)	0 (0-0)	0 (0-1)	0 (0-0)	0 (0-1)	0 (0-0)

*Note.p* < 0.0001 for the difference in mean values between cases and controls for each healthcare service, and across all time periods. Abbreviations: ED, emergency department; GP, general practitioner; IQR, interquartile range; SD, standard deviation. ^†^Not all cases/controls had 24 months of follow-up. ^‡^Cumulative across all inpatient stays within each time period. Data are for number of attendances unless otherwise indicated.

**Table 3:** Healthcare resource utilisation in patients with chronic low back pain + osteoarthritis and matched controls.

Healthcare service use	0–6 months		0–12 months		0–24 months^†^		0–36 months^†^	
	Cases (*N* = 7803)	Controls (*N* = 7803)	Cases (*N* = 7803)	Controls (*N* = 7803)	Cases (*N* = 5406)	Controls (*N* = 5406)	Cases (*N* = 3482)	Controls (*N* = 3482)
*GP consultation*								
Mean (SD)	8.50 (6.30)	3.18 (3.95)	16.23 (11.51)	6.44 (7.22)	30.59 (20.78)	12.74 (12.97)	44.44 (28.58)	19.00 (18.74)
Median (IQR)	7 (4–11)	2 (0–5)	14 (9–21)	4 (2–9)	26 (17–39)	10 (4–17)	38 (25–56)	14 (7–25)

*Outpatient attendance*								
Mean (SD)	4.17 (4.66)	0.93 (2.30)	7.87 (7.72)	1.93 (4.22)	14.07 (12.70)	3.83 (7.44)	19.77 (17.31)	5.70 (10.78)
Median (IQR)	3 (1–6)	0 (0-1)	6 (3–11)	0 (0–2)	11 (6–19)	1 (0–5)	16 (8–26)	2 (0–7)

*ED visit*								
Mean (SD)	0.29 (0.73)	0.10 (0.38)	0.55 (1.20)	0.20 (0.59)	1.03 (2.15)	0.39 (0.94)	1.50 (2.81)	0.55 (1.09)
Median (IQR)	0 (0-0)	0 (0-0)	0 (0-1)	0 (0-0)	0 (0-1)	0 (0-0)	1 (0–2)	0 (0-1)

*Inpatient stay*								
Mean (SD)	0.69 (0.98)	0.17 (1.60)	1.25 (1.63)	0.35 (3.20)	2.22 (2.68)	0.67 (6.11)	3.06 (3.23)	0.96 (8.32)
Median (IQR)	0 (0-1)	0 (0-0)	1 (0–2)	0 (0-0)	2 (1–3)	0 (0-1)	2 (1–4)	0 (0-1)

*Length of stay, days* ^*‡*^								
Mean (SD)	1.32 (5.34)	0.45 (5.80)	2.52 (9.03)	0.90 (7.13)	4.44 (13.19)	1.65 (10.42)	6.05 (18.51)	2.27 (11.38)
Median (IQR)	0 (0–0)	0 (0–0)	0 (0–2)	0 (0–0)	0 (0–4)	0 (0–0)	1 (0–6)	0 (0–0)

*Note.p* < 0.0001 for the difference in mean values between cases and controls for each healthcare service, and across all time periods. Abbreviations: ED, Emergency Department; GP, General practitioner; IQR, interquartile range; SD, standard deviation. ^†^Not all cases/controls had 24 months of follow-up. ^‡^Cumulative across all inpatient stays within each time period. Data are for number of attendances unless otherwise indicated.

## Data Availability

The study data analysed in this publication are derived from the Clinical Practice Research Datalink (https://www.cprd.com) and Hospital Episode Statistics database (https://digital.nhs.uk/data-and-information/data-tools-and-services/data-services/hospital-episode-statistics; copyright © 2022, re-used with the permission of The Health and Social Care Information Centre. All rights reserved). Authors had access to the study data for the purposes of this work only. Data were accessed through an individual dataset study licence to address prespecified research questions only. Therefore, the data cannot be broadly disclosed or made publicly available at this time. Access to each database can be requested via the respective websites.

## Supplementary Materials

Supplementary Materials
*Supplementary Table 1*. Outpatient services used by patients with chronic lower back pain alone and matched controls. *Supplementary Table 2*. Outpatient services used by patients with chronic lower back pain + osteoarthritis and matched controls. *Supplementary Figure 1.* Medicine use across treatment lines in patients with (A) chronic lower back pain alone and (B) chronic lower back pain + osteoarthritis.
