# Supplementary material for: Chronic Low Back Pain with and without Concomitant Osteoarthritis: A Retrospective, Longitudinal Cohort Study of Patients in England
Source: Int J Clin Pract. 2023 Nov 9;2023:5105810. doi: 10.1155/2023/5105810 (PMC10653975; doi:10.1155/2023/5105810)
Supplement: Supplementary Materials — Supplementary Table 1. Outpatient services used by patients with chronic lower back pain alone and matched controls. Supplementary Table 2. Outpatient services used by patients with chronic lower back pain + osteoarthritis and matched controls. Supplementary Figure 1. Medicine use across treatment lines in patients with (A) chronic lower back pain alone and (B) chronic lower back pain + osteoarthritis. [file 5105810.f1.docx]

Supplementary data

**SUPPLEMENTARY TABLE 1** Outpatient services used by patients with chronic lower back pain alone and matched controls

| **Outpatient service use, n (%)^‡^** | **0−6 months** | | **0−12 months** | | **0−24 months^†^** | | **0−36 months^†^** | |
| --- | --- | --- | --- | --- | --- | --- | --- | --- |
|  | **Cases (*N =* 13 554)** | **Controls (*N =* 13 554)** | **Cases (*N =* 13 554)** | **Controls**  **(*N =* 13 554)** | **Cases (*N =* 9728)** | **Controls (*N =* 9728)** | **Cases (*N =* 6724)** | **Controls (*N =* 6724)** |
| Orthopaedics | 4326 (31.9) | 280 (2.1) | 5175 (38.2) | 468 (3.5) | 4244 (43.6) | 576 (5.9) | 3213 (47.8) | 570 (8.5) |
| Pain management | 2693 (19.9) | 19 (0.1) | 3623 (26.7) | 32 (0.2) | 3133 (32.2) | 34 (0.3) | 2382 (35.4) | 32 (0.5) |
| Physiotherapy | 1706 (12.6) | 99 (0.7) | 2344 (17.3) | 187 (1.4) | 2057 (21.1) | 256 (2.6) | 1558 (23.2) | 226 (3.4) |
| Rheumatology | 1145 (8.4) | 147 (1.1) | 1410 (10.4) | 208 (1.5) | 1154 (11.9) | 189 (1.9) | 865 (12.9) | 172 (2.6) |
| Cardiology | 422 (3.1) | 241 (1.8) | 709 (5.2) | 367 (2.7) | 797 (8.2) | 403 (4.1) | 724 (10.8) | 354 (5.3) |
| Gastroenterology | 332 (2.4) | 129 (1.0) | 554 (4.1) | 208 (1.5) | 612 (6.3) | 216 (2.2) | 556 (8.3) | 207 (3.1) |
| Other services | 5636 (41.6) | 2708 (20.0) | 7467 (55.1) | 3835 (28.3) | 6521 (67.0) | 3647 (37.5) | 5057 (75.2) | 3018 (44.9) |

*Note:* All p<0.0001 for cases versus controls.

^†^Not all cases/controls had 24 or 36 months of follow-up. ^‡^Outpatient services were limited to those provided in the secondary care setting only.

**SUPPLEMENTARY TABLE 2** Outpatient services used by patients with chronic lower back pain + osteoarthritis and matched controls

| **Outpatient service use, n (%)^‡^** | **0−6 months** | | **0−12 months** | | **0−24 months^†^** | | **0−36 months^†^** | |
| --- | --- | --- | --- | --- | --- | --- | --- | --- |
|  | **Cases (*N =* 7803)** | **Controls**  **(*N =* 7803)** | **Cases (*N =* 7803)** | **Controls**  **(*N =* 7803)** | **Cases (*N =* 5406)** | **Controls**  **(*N =* 5406)** | **Cases (*N =* 3482)** | **Controls**  **(*N =* 3482)** |
| Orthopaedics | 4378 (56.1) | 184 (2.4) | 5071 (65.0) | 297 (3.8) | 3928 (72.7) | 360 (6.7) | 2697 (77.5) | 333 (9.6) |
| Pain management | 1090 (14.0) | 14 (0.2) | 1466 (18.8) | 23 (0.3) | 1321 (24.4) | 23 (0.4) | 996 (28.6) | 20 (0.6) |
| Physiotherapy | 1139 (14.6) | 85 (1.1) | 1659 (21.3) | 139 (1.8) | 1488 (27.5) | 157 (2.9) | 1119 (32.1) | 163 (4.7) |
| Rheumatology | 692 (8.9) | 134 (1.7) | 896 (11.5) | 178 (2.3) | 768 (14.2) | 165 (3.1) | 580 (16.7) | 135 (3.9) |
| Cardiology | 449 (5.8) | 236 (3.0) | 728 (9.3) | 365 (4.7) | 795 (14.7) | 390 (7.2) | 698 (20.0) | 307 (8.8) |
| Gastroenterology | 243 (3.1) | 101 (1.3) | 392 (5.0) | 155 (2.0) | 429 (7.9) | 164 (3.0) | 403 (11.6) | **1**44 (4.1) |
| Other services | 3987 (51.1) | 2060 (26.4) | 5124 (65.7) | 2821 (36.2) | 4249 (78.6) | 2580 (47.7) | 2974 (85.4) | 1918 (55.1) |

*Note:* All p<0.0001 for cases versus controls.

^†^Not all cases/controls had 24 or 36 months of follow-up. ^‡^Outpatient services were limited to those provided in the secondary care setting only.

**SUPPLEMENTARY FIGURE 1** Medicine use across treatment lines in patients with (A) chronic lower back pain alone and (B) chronic lower back pain + osteoarthritis

A


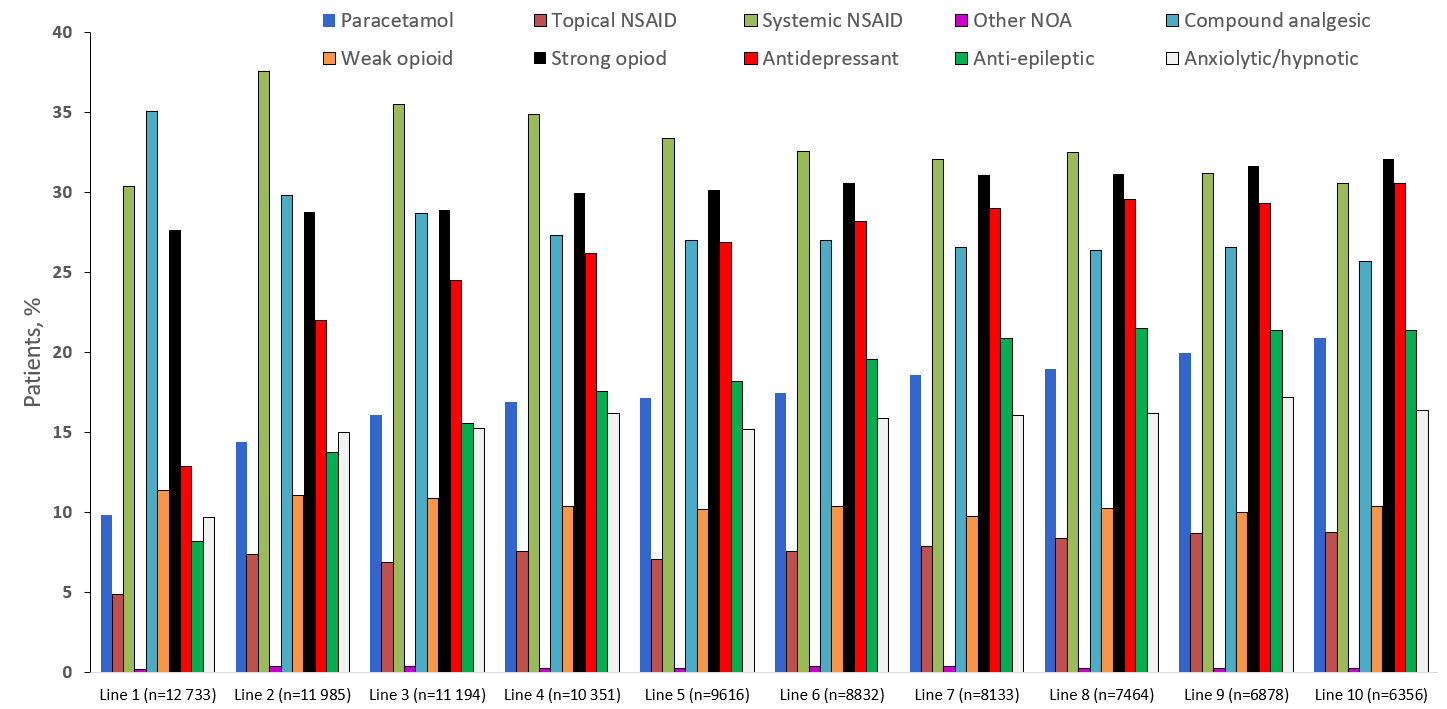


B


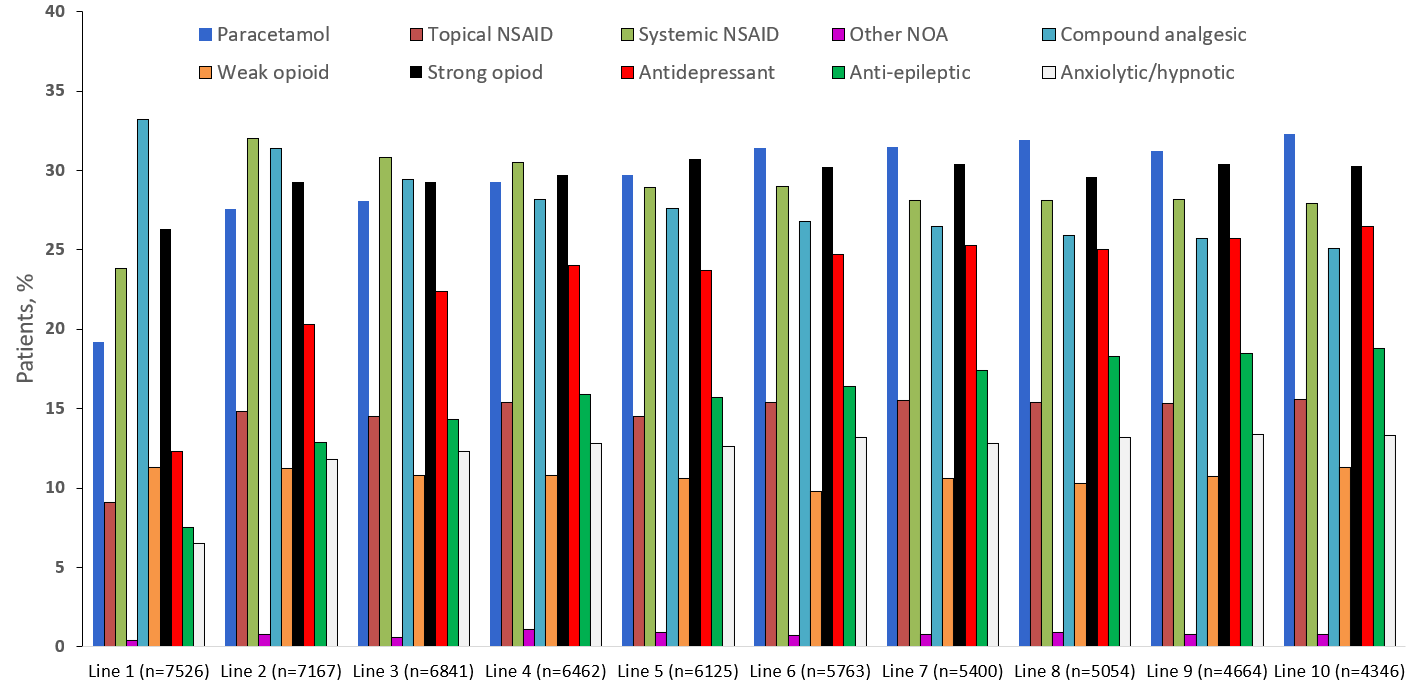


NOA, non-opioid analgesic; NSAID, nonsteroidal anti-inflammatory drug.
